# Organic and inorganic selenium-enriched *Lactobacillus plantarum* differentially enhance bacterial antioxidant capacity and support redox homeostasis in intestinal Caco-2 cells through probiotic and postbiotic effects

**DOI:** 10.3389/fnut.2026.1894526

**Published:** 2026-07-20

**Authors:** Adriana García-Vara, Marta Titos-Zamora, Ruth Ferrer, Raquel Martín-Venegas

**Affiliations:** 1 Departament de Bioquímica i Fisiologia, Facultat de Farmàcia i Ciències de l’Alimentació, Universitat de Barcelona (UB), Barcelona, Spain; 2 Institut de Recerca en Nutrició i Seguretat Alimentària (INSA-UB), Universitat de Barcelona, Santa Coloma de Gramenet, Spain; 3 Departament de Nutrició, Ciències de l’Alimentació i Gastronomia, Facultat de Farmàcia i Ciències de l’Alimentació, Universitat de Barcelona (UB), Barcelona, Spain

**Keywords:** glutathione peroxidase, intestinal antioxidant system, *Lactobacillus plantarum*, postbiotics, probiotics, selenium enrichment, selenomethionine, sodium selenite

## Abstract

Selenium (Se)-enriched probiotics represent a valuable strategy for delivering this essential trace element in both human and animal nutrition. This study evaluated the effects of organic and inorganic Se supplementation at low (5 μM) and high (250 μM) concentrations on the bacterial antioxidant capacity of *Lactobacillus plantarum* (LP), as well as the ability of the resulting probiotics and postbiotics (non-lysed cell-free supernatants, CFS) to modulate redox homeostasis in intestinal Caco-2 cells. Se enrichment did not compromise LP viability and increased glutathione peroxidase (GPx) activity, reduced intracellular reactive oxygen species (ROS) levels, and upregulated *gpx* expression, particularly under high organic Se supplementation. Organic Se also promoted greater intracellular Se accumulation, whereas inorganic Se remained proportionally higher in the extracellular fraction. In Caco-2 cells, both Se-enriched probiotics and postbiotics upregulated *GPX1* expression, with stronger effects observed for whole bacteria than for the CFS. However, no significant changes were observed in GPX enzymatic activity or ROS production in the intestinal cells. In conclusion, the results indicate that Se enrichment modulates Se utilization and antioxidant-related responses in LP, with more pronounced effects observed at higher Se concentrations and under organic Se supplementation. Moreover, both organic and inorganic Se-enriched probiotics and postbiotics modulated *GPX1* expression in Caco-2 cells, supporting their potential as functional ingredients for maintaining intestinal redox homeostasis. This work therefore extends current evidence on Se-enriched LAB and suggests new opportunities for Se-enriched probiotic and postbiotic formulations targeting intestinal redox balance.

## 1 Introduction

The selenium (Se) cycle in the food chain begins in the soil, where it can be found in various chemical forms, both inorganic and organic. Inorganic Se, found primarily in rocks and minerals, occurs in nature in three oxidation states: selenate (SeO₄^2−^), selenite (SeO₃^2−^), and selenide (Se^2−^) (1). Organic sources of Se, less abundant than inorganic ones, include Se analogs of amino acids such as methionine (Met) and cysteine (Cys), namely selenomethionine (SeMet) and selenocysteine (SeCys) (2). The main source of Se for plants is the inorganic form found in the soil (1). Plant roots can rapidly convert soil inorganic Se to organic forms (3, 4). In food, Se is predominantly found in organic forms, primarily as SeMet (5, 6). Over half of the world’s population is at risk of micronutrient deficiencies, with Se deficiency being especially concerning (7, 8). Se intake varies widely across regions due to differences in soil composition, Se speciation, and competing ions that determine the Se concentration in plants and, consequently, in the diets of livestock and humans (2, 9). These variances have major consequences for human and animal health, as they lack the enzymatic capacity to synthesize SeMet *de novo*, and it must be obtained through the diet.

Se is involved in vital metabolic functions, including redox signaling and cellular defense against oxidative stress. These functions are driven by the synthesis of selenoproteins, such as thioredoxin reductase or glutathione peroxidase (GPx), for which Se is a necessary cofactor (10–12). Thus, adequate levels of Se in humans and animals protect against lipid peroxidation, the accumulation of compounds containing carbonyl groups, and protein fragmentation caused by oxidative stress (11). Although selenoproteins are generally classified as antioxidants, they also regulate thyroid hormone metabolism, immune and inflammatory responses (10, 13–15). Proper Se intake has been linked to reduced risks of cancer, cardiovascular diseases, diabetes, and infertility (16–18). Inadequate dietary Se is particularly relevant in the gut, where it is considered a risk factor for chronic diseases associated with oxidative stress and inflammation (19–21), making Se a potential therapeutic agent. However, ensuring sufficient Se intake through diet remains challenging, motivating the exploration of Se-enriched foods and functional supplements. Se is increasingly recognized as an important modulator of gut microbiota composition and function (22). Dietary supplementation enhances microbial diversity and shapes gastrointestinal colonization patterns, emphasizing the unique role in microbiota dynamics (23, 24). Notably, approximately 20% of gut microorganisms possess the capacity to express selenoproteins, and their expression is directly regulated by Se availability (23). Similar to plants, many bacteria and yeast can convert inorganic Se into organic forms (15, 25). Several strains of *Lactobacillus* and *Bifidobacterium* have demonstrated the ability to bio-transform inorganic Se into SeCys and/or SeMet (26–30), enabling its use as a host accessible Se source. However, under conditions of limited Se availability, competition may occur, potentially reducing Se accessibility for host selenoprotein synthesis (23, 31). Thus, the microbiota can modulate the host Se status. Furthermore, Se can exhibit dose and form dependent toxicity toward certain microbial groups, adding another layer of complexity to Se-microbiota interactions (32, 33). Despite these insights, the interaction between trace elements, microbial metabolism, and host antioxidant defenses remains only partially understood, highlighting the need for further research into Se’s complex roles in the gut environment.

Probiotics have been widely investigated for their capacity to modulate gut homeostasis and confer health benefits through multiple mechanisms. These include the maintenance of microbial balance, competitive exclusion of pathogens, production of antimicrobial compounds, and modulation of host immune responses (34–36). In addition, certain probiotic strains can enhance intestinal barrier integrity by upregulating tight junction proteins and stimulating mucus production, thereby reducing gut permeability (34, 37). Probiotics are also known to contribute to host antioxidant defenses by producing metabolites such as short-chain fatty acids and by influencing redox signaling pathways (38, 39). *Lactobacillus plantarum* (now reclassified as *Lactiplantibacillus plantarum*) is one of the most common strains used in probiotic preparations (34) and it has been described to be able to tolerate and accumulate Se (8, 35), making it a good candidate as a Se-enriched probiotic. This represents a different method to boost Se intake, offering an alternative to the traditional use of inorganic Se to produce a wide variety of Se-enriched products such as milk, eggs, and meat (1, 40, 41), with the potential to enhance antioxidant defenses, reinforce tight junction proteins, and improve intestinal barrier function (35, 36). Also, studies regarding mechanisms involved in the probiotic effect revealed that it is partially due to cell parts or metabolites released by probiotic microorganisms (42). This led to the introduction of the term “postbiotics” in 2013, defined as “any factor resulting from the metabolic activity of a probiotic or any released molecule capable of conferring beneficial effects to the host in a direct or indirect way” (43). Recently, the International Scientific Association for Probiotics and Prebiotics (ISAPP) stated that “postbiotics are preparations of inanimate microorganisms and/or their components that confer health benefits” (37, 38). Postbiotic-derived *L. plantarum* has shown antioxidant activity, gut barrier modulation, antimicrobial effect, and other health benefits (39, 44–46). Lactic acid bacteria (LAB) strains, generally recognized as safe (GRAS), when cultured in Se-supplemented media, can also accumulate organic Se compounds. A promising yet underexplored path is the development of next-generation functional products, such as mineral-enriched postbiotics. These formulations could be incorporated into fermented foods such as yogurt, sourdough bread, pickles, and fruit juices, potentially offering additional benefits like improved antioxidant capacity (4, 28, 47) and provide essential minerals (e.g., calcium, magnesium, iron, zinc, and Se) in highly bioavailable forms while also delivering the health benefits of postbiotic components (48). Se-enriched microorganisms and fermented products are emerging strategies, compared to common Se supplements or additives, to improve Se bioavailability, while providing additional health benefits (47, 49–51), although their effects on intestinal epithelial antioxidant defenses remain poorly understood.

Current research on Se-enriched LAB has primarily focused on the bioaccumulation and biotransformation of inorganic Se sources, as well as their bioavailability for host selenoprotein synthesis. This emphasis is partly explained by the widespread use of inorganic Se in enrichment studies, given its lower cost and its ability to be converted into organic Se forms by bacteria. However, in human diets, Se is predominantly consumed in organic forms, such as SeMet, which can directly interact with the gut microbiota. Despite their physiological relevance, the effects of organic Se sources on bacterial physiology, viability, and antioxidant defenses remain poorly understood. Likewise, most studies investigating the biological effects of Se-enriched microorganisms have focused on bacterial biomass or cell lysates, making it difficult to distinguish the contribution of intracellular Se from that of the secreted microbial metabolites. As a result, the biological activity of non-lysed cell-free supernatants (CFS, or postbiotics) from Se-enriched LAB remains largely unexplored.

To address these gaps, we adopted an integrated approach to evaluate both the probiotic and postbiotic potential of Se-enriched *L. plantarum* on intestinal Caco-2 cells. First, we compared the effects of organic and inorganic Se sources on bacterial viability and antioxidant capacity across different Se concentrations. We then investigated how Se-enriched *L. plantarum*, incubated either as live bacteria (probiotic) or as CFS (postbiotic), modulates Se-dependent selenoprotein expression and oxidative stress in Caco-2 intestinal cells. By directly comparing organic and inorganic Se sources and simultaneously assessing the activity of both probiotic and postbiotic preparations, this study provides new insight into how dietary Se forms are processed by LAB and how both bacterial cells and their secreted metabolites contribute to intestinal redox regulation. These findings may support the development of more targeted Se-enriched probiotic and postbiotic strategies aimed at promoting gut redox homeostasis.

## 2 Materials and methods

### 2.1 Materials

Seleno-L-methionine (SeMet), sodium selenite (SS, Na₂SeO₃), Dulbecco’s modified Eagle’s medium (DMEM), Dulbecco’s phosphate-buffered saline (DPBS) and TRI Reagent were obtained from Sigma-Aldrich (St. Louis, MO, United States). Fetal bovine serum (FBS) was purchased from Cytiva (Marlborough, MA, United States). De Man, Rogosa, and Sharpe (MRS) agar was obtained from Thermo Fisher Oxoid (Hampshire, United Kingdom). Kits for GPx activity were purchased from Abcam (Cambridge, United Kingdom). RNA extraction and reverse transcription kits were from Thermo Fisher Scientific (Waltham, MA, United States). Tissue culture supplies were obtained from Costar (Cambridge, MA, United States).

### 2.2 Bacterial culture and Se supplementation

*Lactobacillus plantarum* ATCC 8014 (LP) was kindly provided by Dr. Ana Marqués (Facultat de Farmàcia i Ciències de l’Alimentació, Universitat de Barcelona) and prepared as previously described (52). Bacteria were grown on MRS agar at 37 °C for 48 h. Cultures in the exponential phase (Optical density, OD₆₀₀ = 0.8–1.0, corresponding to 7·10^7^–3 ·10^8^ CFU/mL) were used for experiments. Five conditions were tested: LP without Se (LP0), and LP supplemented at two different concentrations (5 and 250 μM) of SeMet (LP-SM5 and LP-SM250) or of SS (LP-SS5 and LP-SS250). For the viability assay, LP conditions were cultured on MRS agar plates. All the remaining experiments were carried out in flasks or wells in DMEM supplemented with 1% (v:v) non-essential amino acids, 10% (v:v) heat-inactivated FBS in a modified atmosphere of 5% CO2 in air at 37 °C.

### 2.3 Preparation of CFS

For the preparation of CFS, bacterial cultures were incubated for 36 h under the conditions described at section 2.2 in 6-well plates (2·10^7^ CFU/mL). Cells were harvested by centrifugation (10,000 × g, 5 min, 4 °C), resuspended in standard DMEM, and incubated for an additional 12 h (total 48 h). Non-lysed supernatants were collected by centrifugation (16,000 × g, 10 min, 4 °C) and adjusted with antibiotic-containing DMEM (final 50% v:v) (53). Standard DMEM without bacterial inoculation served as a negative control.

### 2.4 Effect of Se sources on LP

#### 2.4.1 Growth and viability assays

MRS agar plates previously supplemented with SeMet or SS (5 μM or 250 μM) were inoculated with a LP suspension (80 CFU/mL) and incubated at 37 °C for 48 h as previously described (52). CFUs were counted at 48 h and 96 h. MRS agar without Se supplementation was used as a control. In a complementary assay, LP cultures (8·10^6^ CFU/mL) were incubated in 25 cm^2^ flasks under the conditions described in section 2.2 for 96 h at 37 °C. OD₆₀₀ was measured every 24 h to construct growth curves. LP cultures were observed in an Olympus BX41 microscope equipped with a camera (Hamburg, Germany) and images were taken with a 40x objective lens. To evaluate a possible cytotoxic effect of these sources, lactate dehydrogenase (LDH) release was analyzed in the culture medium as previously described (54).

#### 2.4.2 GPx activity assay in LP

Cultures adjusted to 2·10^7^ CFU/mL were incubated in 6-well plates as described in section 2.2 for 48 h. Bacteria were collected by centrifugation (10,000 × g, 5 min, 4 °C), resuspended in 200 μL cold DPBS, and disrupted by ultrasonic oscillation (4 cycles, 15 s each, probe tip 1.27 cm, <4 °C). Lysates were centrifuged (10,000 × g, 5 min, 4 °C), and supernatants were stored at −80 °C. GPx activity was determined using the Glutathione Peroxidase Activity Assay Kit (Fluorometric) (Abcam, United Kingdom), following the manufacturer’s protocol. Absorbance was recorded at 420/480 nm in kinetic mode for 60 min (Varioskan LUX Multimode Microplate Reader, Thermo Fisher Scientific). Total protein content was determined by spectrophotometry, using the protein determination protocol from Varioskan LUX Multimode Microplate Reader. Activity was normalized to total protein content (U/mg protein) before being converted to a percentage.

#### 2.4.3 *gpx* gene expression in LP

Cultures adjusted to 2·10^7^ CFU/mL were incubated in 6-well plates as described in section 2.2 for 48 h. For gene expression, samples were collected by centrifugation (10,000 × g, 5 min, 4 °C). Pellets were stored at −80 °C until RNA extraction. Total RNA was isolated using TRIzol™ Max™ Bacterial RNA Isolation Kit (Thermo Fisher). RNA concentration was determined with a Varioskan LUX Multimode Microplate Reader (Thermo Fisher). Reverse transcription was performed with High-Capacity RNA-to-cDNA Kit (Life Technologies), and cDNA was analyzed by real-time quantitative PCR (qPCR).

*16S* ribosomal RNA gene was used as the reference, based on previous literature (55). Primers for *gpx* were designed from the *L. plantarum* ATCC 8014 genome (GenBank accession NZ_CP024413) using Primer-BLAST (NIH). Custom TaqMan assays (Thermo Fisher) were used for qPCR. Data were analyzed by the ∆∆Ct method, with expression levels normalized to the untreated control (set at 100%). qPCR was performed at Genomic Unit, Centres Científics i Tecnològics, Universitat de Barcelona. Design & Analysis Software 2.6.0 was used to analyze the data.

#### 2.4.4 Intracellular ROS assay in LP

Cultures adjusted to 2·10^7^ CFU/mL were incubated in 6-well plates as described in section 2.2 for 48 h. Intracellular ROS production was assessed using the oxidation of 2′,7′-dichlorofluorescein diacetate (DCF-DA) to its fluorescent derivative, dichlorofluorescein (DCF). The assay was performed with a commercial intracellular ROS detection kit (OxiSelect; Cell Biolabs Inc.) according to the manufacturer’s protocol. Briefly, bacteria were treated with 0.1% Triton X-100 for 10 min and subsequently washed with DPBS. Samples were then incubated at 37 °C with 2′,7′-dichlorofluorescein (100 μmol/L in DMEM) for 45 min under reduced-light conditions. After incubation, the bacterial pellet was washed with antibiotic-free DMEM to remove any unincorporated probe. Fluorescence intensity was recorded at the start and end of the incubation period using a Varioskan LUX Multimode Microplate Reader (Thermo Fisher Scientific) with excitation and emission wavelengths set to 480 nm and 535 nm, respectively.

#### 2.4.5 Se quantification

Se was quantified across all five supplemented conditions in each of the following sample fractions: (a) supplemented DMEM, (b) bacterial pellets collected by centrifugation (10,000 × g, 5 min, 4 °C) after 48 h of incubation or (c) CFS of supplemented bacteria cultures. ICP-MS (Inductive Coupled Plasma Mass Spectrometry) analyses were carried out by the Metal Analysis Unit, Centres Científics i Tecnològics (CCiTUB), Universitat de Barcelona.

### 2.5 Effect of Se-enriched LP and CFS on Caco-2 cells

#### 2.5.1 Caco-2 cell culture and co-incubation with LP or CFS

Caco-2 cells were obtained from the European Collection of Authenticated Cell Cultures. Cells were maintained in DMEM supplemented with 1% (v:v) non-essential amino acids, 10% (v:v) heat-inactivated FBS, 100 UI/ml penicillin and 100 μg/mL streptomycin at 37 °C in a modified atmosphere of 5% CO_2_. Cells were subcultured at a density of 8·10^3^ cells/cm^2^ as previously described (56). Differentiated monolayers (15 days in culture, passages 60–80) were seeded in 6-well plates for GPX activity and *GPX1* gene expression assay and in 24-well plates for ROS assay.

Differentiated Caco-2 monolayers were washed with DMEM without antibiotic and incubated with LP under the conditions described in section 2.2 at a multiplicity of infection (MOI) of 10 or CFS (50% v:v) for 4 h at 37 °C. After incubation, cells were washed twice with cold DPBS to remove LP or CFS.

#### 2.5.2 GPX activity in Caco-2 cells

Cells were scraped into 1 mL cold DPBS and disrupted by ultrasonic oscillation (2 cycles, 15 s each, <4 °C). Lysates were centrifuged (10,000 × g, 5 min, 4 °C), and supernatants were stored at −80 °C. GPX activity was measured using the commercial Glutathione Peroxidase Activity Assay Kit (Fluorometric) (Abcam, United Kingdom) as previously described (19).

#### 2.5.3 *GPX1* gene expression in Caco-2 cells

Total RNA was extracted with TRI Reagent (Life Technologies). RNA concentration was determined spectrophotometrically, and reverse transcription was performed using the High Capacity RNA to cDNA Kit (Life Technologies) following the manufacturer’s recommendations, in a thermal cycler (Thermomixer comfort, Eppendorf) at 37 °C for 60 min, then reactions were inactivated at 95 °C for 5 min. *GPX1* expression was quantified by qPCR using pre-designed TaqMan Gene Expression Assays (*GPX1*_glutathione peroxidase 1, Life Technologies) as previously described (57). Among tested reference genes (*ACTB_β-actin, GAPDH_* glyceraldehyde-3-phosphate dehydrogenase*, RPLP0_* ribosomal Protein Lateral Stalk Subunit P0), *ACTB* was selected for normalization.

#### 2.5.4 Intracellular ROS assay in Caco-2 cells

Intracellular ROS generation was assessed by the intracellular oxidation of 2′,7′-dichlorofluorescein to the fluorescent compound dichlorofluorescein and performed with the use of a commercial intracellular ROS assay kit (OxiSelect; Cell Biolabs Inc.) following the manufacturer’s instructions. Before inoculation with LP or CFS, Caco-2 monolayers were washed with DPBS and incubated at 37 °C with 2′,7′-dichlorofluorescein (100 μmol/L in DMEM) for 40 min in attenuated light conditions. Monolayers were then washed twice with antibiotics-free DMEM to remove excess probe. Fluorescence intensity was measured at the beginning and end of incubation using a Varioskan LUX Multimode Microplate Reader at excitation/emission wavelengths of 480/535 nm as previously described (19, 54).

### 2.6 Statistical analysis

Data are expressed as mean ± SD. Statistical analysis was performed using one-way ANOVA followed, when appropriate, by Bonferroni’s *post hoc* test. Comparisons between bacteria and CFS were performed using Student’s *t*-test. Analyses were conducted using IBM SPSS Statistics version 29 (SPSS Inc., Chicago, IL, United States), and a *p*-value < 0.05 was considered statistically significant.

## 3 Results

### 3.1 Effect of Se supplementation on LP viability and growth curve

**Figure 1:**
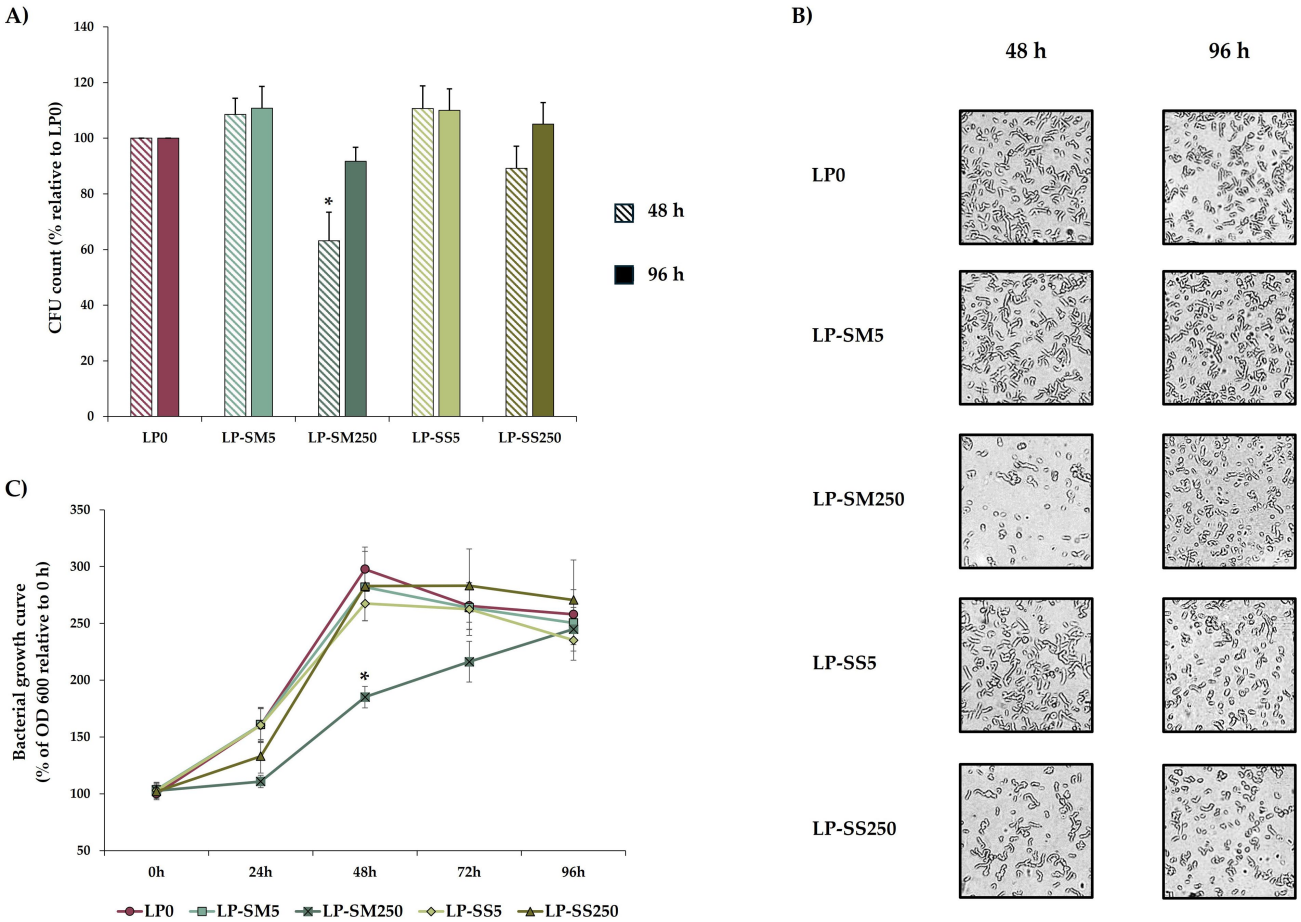
Viability and LP growth after Se enrichment. **(A)** Viability was assessed by colony-forming unit (CFU) counts in MRS plates enriched with Se sources (*n* = 6). **(B)** Representative images of LP growth in DMEM supplemented with Se sources at 48 and 96 h. **(C)** Growth curves of LP in DMEM enriched with Se sources measured by OD_600_ over 96 h (*n* = 4). LP0, LP without Se; LP-SM5 and LP-SM250, LP supplemented with SeMet at 5 or 250 μM, respectively; LP-SS5 and LP-SS250, LP supplemented with SS at 5 or 250 μM, respectively. Data are presented as mean ± SE. * Indicate difference respect to LP0 (*p* < 0.05).

**Figure 2:**
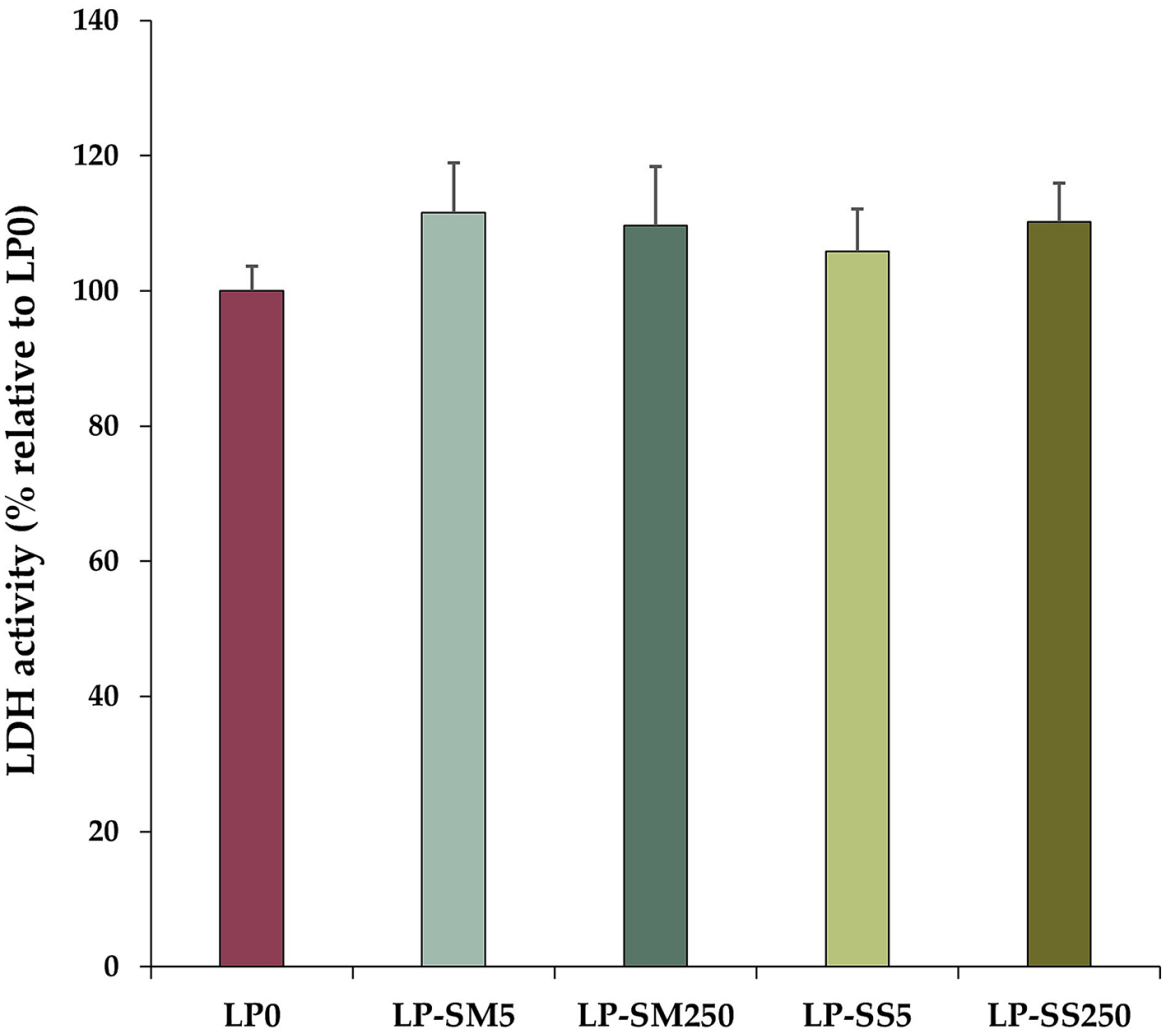
Lactate dehydrogenase (LDH) activity in LP after Se enrichment for 48 h. LP0, LP without Se; LP-SM5 and LP-SM250, LP supplemented with SeMet at 5 or 250 μM, respectively; LP-SS5 and LP-SS250, LP supplemented with SS at 5 or 250 μM, respectively. Data are presented as mean ± SE (*n* = 9). No significant differences were observed between groups (*p* > 0.05).

To ensure that Se enrichment did not compromise the integrity of LP, viability was assessed (Figure 1A). At 48 h, CFU values for the low-concentration groups (LP-SM5 and LP-SS5) remained comparable to LP0. In contrast, LP-SM250 resulted in a significant reduction of bacterial counts (*p* < 0.05). However, by 96 h, LP-SM250 showed similar results to the control, and no significant differences in viability were detected among any experimental groups. These results are accompanied by a visual confirmation in the images of Figure 1B. To further characterize these growth dynamics, a kinetic growth curve was established over a 96 h period (Figure 1C). All groups exhibited a similar growth pattern, with the exception of LP-SM250, which showed a significantly lower values at 48 h compared with the control (*p* < 0.05). However, as we observed in the CFU count, by 96 h OD600 values were similar to the other groups, indicating a recovery in growth. To determine if the decrease in growth at 48 h was due to toxicity, LDH was measured (Figure 2). All Se-supplemented groups (LP-SM5, LP-SM250, LP-SS5, and LP-SS250) exhibited similar relative LDH activity compared to the control (LP0) (*p* > 0.05). These results confirm that Se enrichment does not induce toxicity in LP at these concentrations but rather induces a growth delay under the LP-SM250 condition. Accordingly, all following experiments were performed at 48 h, where control culture attain the plateau.

### 3.2 Impact of Se supplementation on the antioxidant capacity of LP

**Figure 3:**
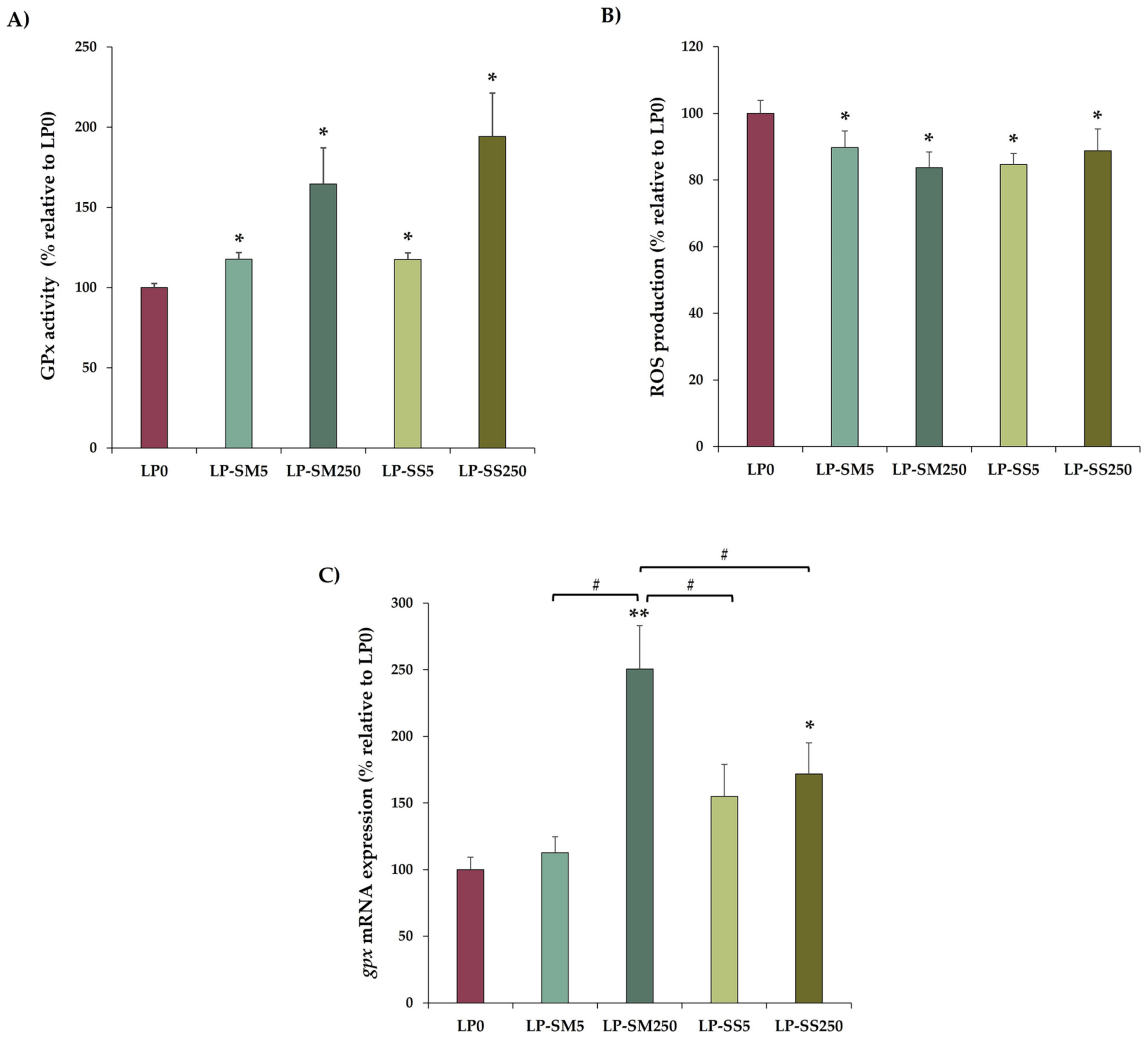
Antioxidant capacity of LP after Se enrichment for 48 h. **(A)** GPx activity, **(B)** ROS production, and **(C)** *gpx* mRNA expression. LP0, LP without Se; LP-SM5 and LP-SM250, LP supplemented with SeMet at 5 or 250 μM, respectively; LP-SS5 and LP-SS250, LP supplemented with SS at 5 or 250 μM, respectively. Data are presented as mean ± SE (*n* = 6). * Indicates significant differences compared to LP0 (*p* < 0.05) and ** (*p* < 0.001). # Indicates differences between Se conditions (*p* < 0.05).

Enzymatic GPx activity increased significantly across all Se-enriched conditions compared to the LP0 (*p* < 0.05; Figure 3A), showing higher values as Se concentrations increased. Consistent with these results, the activation of the GPx system was accompanied by a significant decrease in ROS levels across all treated groups (*p* < 0.05; Figure 3B). Gene expression analysis indicated that Se enrichment upregulated *gpx* in a concentration-dependent manner (Figure 3C). While low concentrations (5 μM) showed similar values to LP0, high concentrations treatments (250 μM) increased *gpx* gene expression significantly. LP-SM250 exhibited the highest expression levels, with *gpx* mRNA levels more than double than LP0 (*p* < 0.001) and significantly higher than those observed with the inorganic form (*p* < 0.05).

### 3.3 Se quantification

The efficiency of Se uptake and its distribution between the extracellular and intracellular compartments were evaluated across the different treatment groups (Table 1). Analysis of the initial medium confirmed that Se concentrations in the supplemented groups were consistent with the target doses of 5 and 250 μM, without differences between sources but showing significant differences relative to the basal levels in the LP0 control (*p* < 0.05). After enrichment, bacterial pellets show significant differences between control (LP0) and all treatment groups. Intracellular Se accumulation in the bacterial pellet was markedly higher for the organic source (SM), showing approximately 12-fold greater uptake than LP-SS5 and 2-fold greater than LP-SS250 (*p* < 0.05), with significant differences also observed between the two concentrations. In the extracellular fraction (CFS), Se content differed significantly between concentrations but showed no significant differences between Se sources. However, when evaluating Se partitioning via the CFS-to-pellet ratio, a clear distinction between sources emerged at the lowest concentration: the proportion of extracellular Se relative to intracellular Se was significantly higher for the inorganic source (SS) than for the organic source (SM) (*p* < 0.05). Additionally, a pink coloration was observed in the pellet of LP-SS250 after 48 h of incubation.

**Table 1:** Total Se content in different fractions after Se enrichment for 48 h.

Variable	LP0	LP-SM5	LP-SM250	LP-SS5	LP-SS250
Initial medium (μg)	0.010 ± 0.001^c^	1.005 ± 0.009^b^	58.002 ± 0.173^a^	0.793 ± 0.039^b^	52.755 ± 3.012^a^
Pellet (μg)	0.002 ± 0.000^e^	0.690 ± 0.110^c^	5.664 ± 0.195^a^	0.057 ± 0.002^d^	2.581 ± 0.501^b^
CFS (μg)	0.014 ± 0.003^b^	0.038 ± 0.006^b^	0.317 ± 0.049^a^	0.027 ± 0.010^b^	0.224 ± 0.049^a^
CFS*100/Pellet (%)	–	5.5 ± 0.1^b^	5.7 ± 1.0^b^	46.3 ± 16.4^a^	10.6 ± 4.8^b^
Pellet color	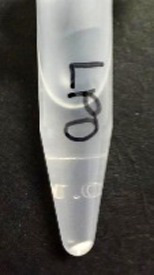	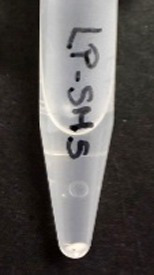	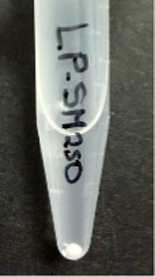	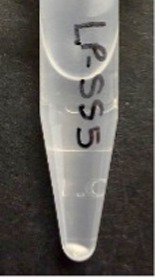	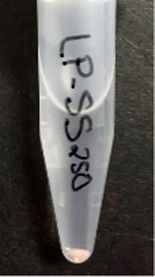

Se content in supplemented initial growth medium correspond to 5 and 250 μM. LP0, LP without Se; LP-SM5 and LP-SM250, LP supplemented with SeMet at 5 or 250 μM, respectively; LP-SS5 and LP-SS250, LP supplemented with SS at 5 or 250 μM; CFS, cell free supernatant. Data are presented as mean ± SE of three individual experiments. Different letters in the same row indicate significant difference between conditions (*p* < 0.05).

### 3.4 Modulation of antioxidant capacity in Caco-2 cells by Se-enriched LP and CFS

**Figure 4:**
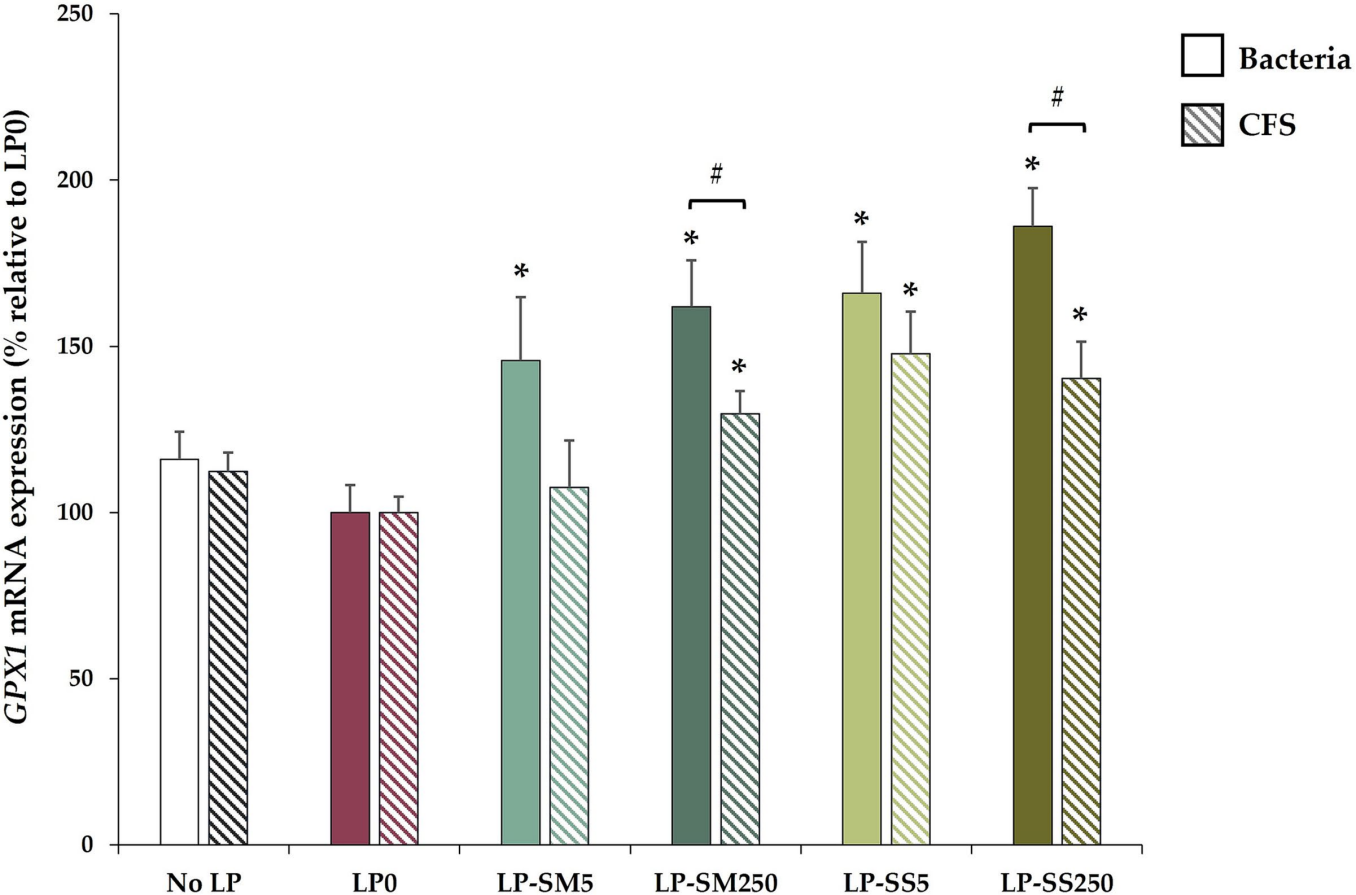
*GPX1* mRNA expression in Caco-2 cells after Se-enriched LP (Bacteria, solid bars) or CFS (diagonal hatched bars). No LP, untreated Caco-2 cells; LP0, cells treated with non-enriched LP or CFS; LP-SM5 and LP-SM250, cells treated with LP or CFS enriched with SeMet at 5 or 250 μM, respectively; LP-SS5 and LP-SS250, cells treated with LP or CFS enriched with SS at 5 or 250 μM, respectively. Data are presented as mean ± SE (*n* = 10). * Indicates significant differences compared to the respective LP0 (*p* < 0.05). # Indicates difference between LP and CFS (*p* < 0.05).

**Figure 5:**
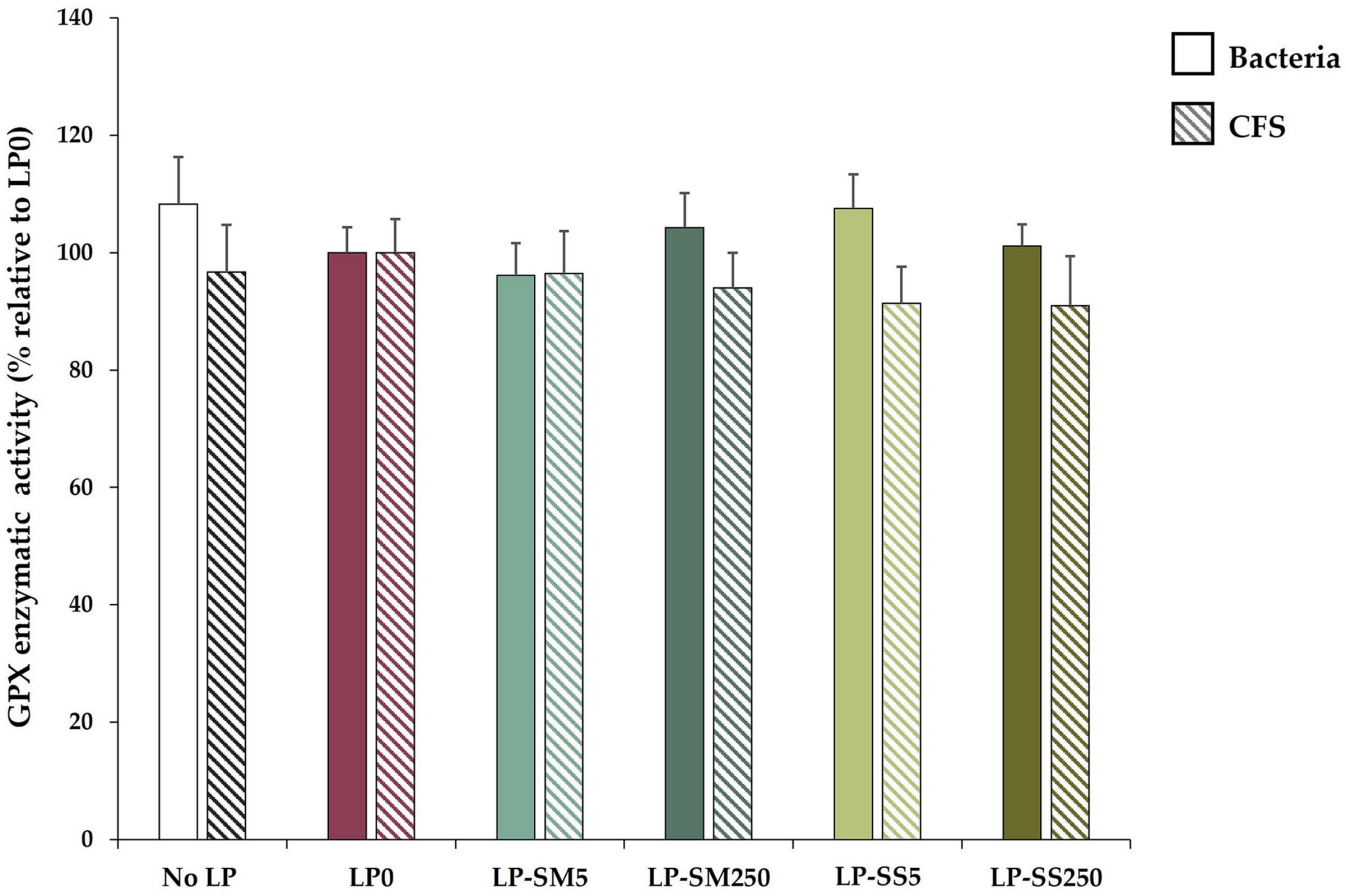
GPX enzymatic activity in Caco-2 cells after Se-enriched LP (Bacteria, solid bars) or CFS (diagonal hatched bars). No LP, untreated Caco-2 cells; LP0, cells treated with non-enriched LP or CFS; LP-SM5 and LP-SM250, cells treated with LP or CFS enriched with SeMet at 5 or 250 μM, respectively; LP-SS5 and LP-SS250, cells treated with LP or CFS enriched with SS at 5 or 250 μM, respectively. Data are presented as mean ± SE (*n* = 6). No significant differences were observed between groups (*p* > 0.05).

**Figure 6:**
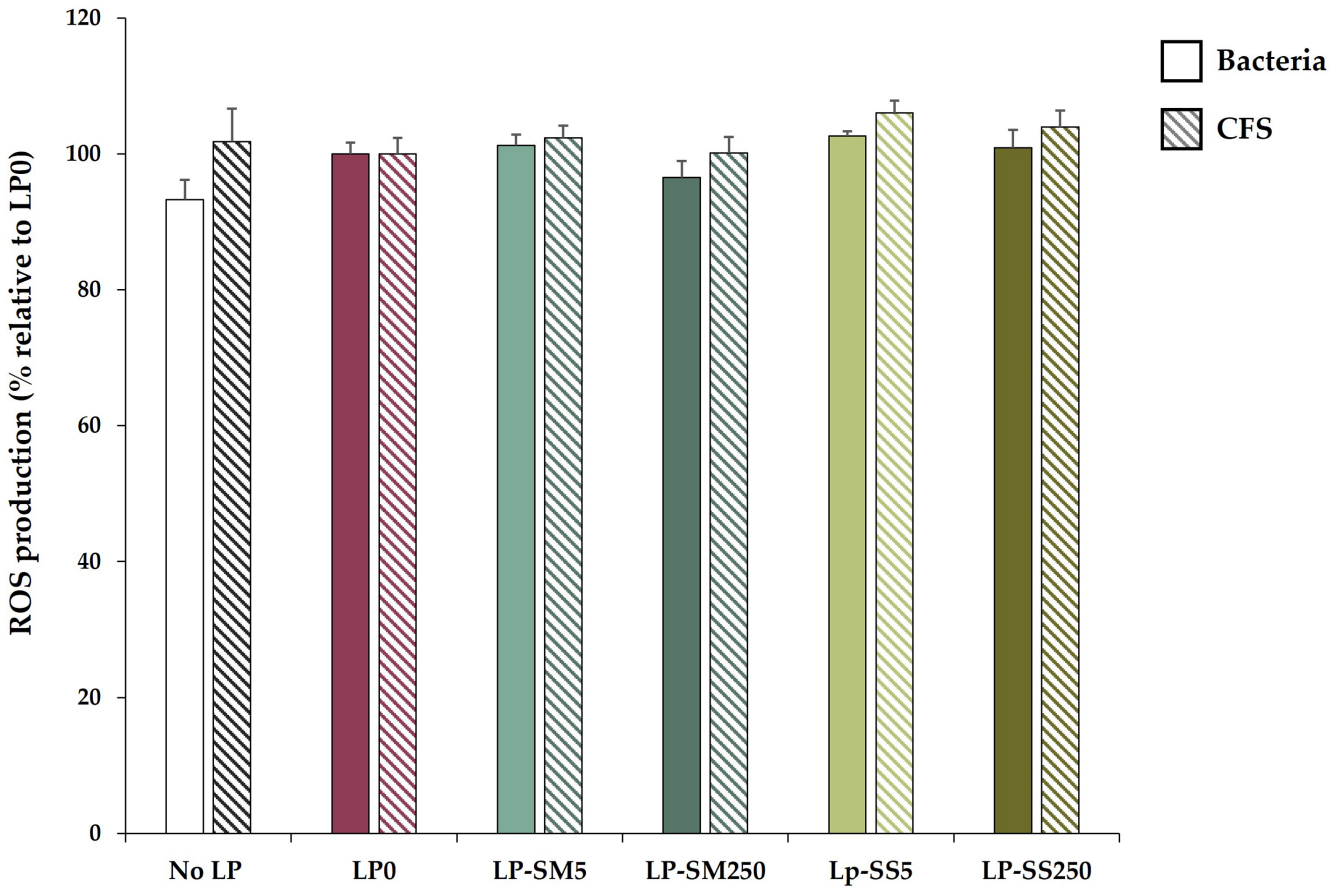
ROS production in Caco-2 cells after Se-enriched LP (Bacteria, solid bars) or CFS (diagonal hatched bars). No LP, untreated Caco-2 cells; LP0, cells treated with non-enriched LP or CFS; LP-SM5 and LP-SM250, cells treated with LP or CFS enriched with SeMet at 5 or 250 μM, respectively; LP-SS5 and LP-SS250, cells treated with LP or CFS enriched with SS at 5 or 250 μM, respectively. Data are presented as mean ± SE (*n* = 6). No significant differences were observed between groups (*p* > 0.05).

The untreated Caco-2 negative controls (No LP and CFS-No LP) showed no difference in relative *GPX1* mRNA expression compared with the non-enriched bacterial controls (LP0 or CFS-LP0). However, all Se-supplemented groups exhibited a significant increase expression compared with the non-enriched controls (*p* < 0.05; Figure 4). Similar trends were observed in the CFS treatments, with the exception of CFS-LP-SM5, which did not show a significant increase. Moreover, for the highest concentration, the relative upregulation was higher in cells treated with whole bacteria than in those treated with supernatants. In contrast, functional GPX enzymatic activity remained unchanged following treatment with either Se-enriched LP or CFS (*p* > 0.05) (Figure 5). Consistent with these findings, ROS production remained stable across all groups treated with whole bacteria or supernatant, with no statistical differences (*p* > 0.05) (Figure 6). Furthermore, negative controls (No LP) showed no significant difference from LP0.

## 4 Discussion

Se is an essential micronutrient, and its deficiency is associated with various diseases, as it plays a key role in protecting cells against inflammation and oxidative stress. In our previous studies, we demonstrated that supplementation with Se sources upregulated the expression of selenoproteins in Caco-2 cells and macrophages (19, 20), thus confirming that Se contributes to the antioxidant and anti-inflammatory functions of the intestinal barrier. However, one crucial component of the intestinal barrier that remains less explored is the microbiota. In this study, we investigated the effects of Se supplementation on LP, as well as the probiotic and postbiotic effects on a Caco-2 cell model, with a particular focus on comparing the use of organic and inorganic Se sources.

The enrichment of microorganisms with Se has been widely studied due to their ability to convert inorganic Se into organic forms, which has potential applications as dietary supplements, food additives, or components of fermented foods (6, 35, 58–60). However, most studies have focused on supplementation with inorganic Se sources. Our quantification of Se in LP-enriched bacteria confirmed Se presence in both the bacterial pellet and the CFS for both sources, indicating that the bacteria effectively internalize and excretes Se. Nevertheless, Se distribution between intracellular and extracellular compartments revealed a clear difference in how LP uses organic versus inorganic sources. Although Se accumulation occurred in all treatment groups, as shown in the pellet content, the higher accumulation in the SeMet-treated bacteria highlights the superior bioavailability of organic Se. This is likely attributable to SeMet’s similarity to Met and its ability to use bacterial Met transport systems to enter the cell (30, 37, 59). In contrast, inorganic Se uptake typically occurs via less specific pathways; for selenate or selenite, uptake has been reported via non-specific phosphate or sulfate transporters, oxyanion transporter proteins, or through passive diffusion across the membrane (61, 62).

Moreover, SeMet can be incorporated into the Met pool and can be non-specifically inserted into proteins, acting as an endogenous reservoir that accumulates in tissues and is slowly released during protein turnover (29, 59, 63–66). Consistently, the high intracellular concentration of SeMet suggests that LP can store organic Se. In contrast, inorganic forms are described to be rapidly reduced to selenide and enter directly into the metabolic pool, without significant storage (66, 67). This is supported by the higher CFS-to-pellet ratio observed under SS conditions compared to SeMet, suggesting that SS is predominantly excreted. High concentrations of selenite have been reported to trigger a detoxification state, in which selenite is reduced to elemental Se (Se^0^) or biogenic Se nanoparticles (SeNPs), which are subsequently excreted or deposited on the cell membrane to mitigate toxicity (37, 62). This aligns with observations by Andreoni et al. (67), who noted that *Lactobacillus* strains exposed to selenite (126 μM for 16 h) generated Se^0^ deposits on the cell surface. These authors also reported the appearance of a pinkish color as a detoxification mechanism under high Se concentrations, which we observed at the highest SS concentration. Inorganic Se can also be converted into selenophasphate, which is a substrate for SeCys and/or SeMet synthesis (25, 26, 68). Our results suggest that the conversion to organic forms by LP is emphasized at higher concentrations (LP-SS250). In this condition, we observed lower CFS-to-pellet ratio compared to LP-SS5, thus confirming that Se is being accumulated.

Effective Se-enrichment of probiotics requires the conversion of inorganic Se into organic forms without adverse effects on cellular structure or physiology. Our results show a reduction in bacterial growth after 48 h of exposure to high SeMet concentration. Previous studies have reported that high Se concentrations can negatively affect bacterial growth and viability (33, 63, 69, 70). Plateau et al. (71), demonstrated that SeMet toxicity arises from its metabolic transformation into SeCys, which could be then indiscriminately incorporated into proteins in place of cysteine, triggering protein aggregation and superoxide production. This can temporarily slow down cellular metabolism until the cells activate adaptive mechanisms to restore homeostasis (59, 63, 72), explaining the lower growth curve observed in LP-SM250 condition. However, a recovery phase was observed at 96 h, indicating that LP was able to adapt and maintain its bioavailability and structural integrity. This adaptive response is consistent with previous observations showing that *Lactobacillus* can modulate Se uptake (67, 73). The temporary growth delay observed at high SeMet levels in LP likely reflects the same stress adaptation balance described for *Rhodobacter sphaeroides* and *Rhodobacter rubrum*, where elevated selenite causes pronounced growth inhibition and reduced stationary phase densities but does not necessarily decrease final cell numbers, as cells divert resources to counteract oxidative and metabolic stress (63, 70, 74, 75). The absence of increased LDH activity in the treatment groups supports that Se enrichment does not compromise membrane integrity or induce acute cytotoxic damage, indicating good biocompatibility of both Se sources.

Se enrichment directly facilitates the synthesis of active GPx by supplying the limiting cofactor, as SeCys constitutes the essential active site of selenoproteins, most notably GPx (74, 76). In agreement with this, our results show an increase in GPx enzymatic activity in LP following Se enrichment, accompanied by a substantial reduction in ROS, confirming the maintenance of redox homeostasis. Notably, this effect was observed for both organic and inorganic Se sources, indicating a similar capacity to reinforce the cellular antioxidant bacterial defense system. The increase in GPx activity observed in our results aligns with the findings of Chen et al. (73), who reported that incubation with SS (50.7–101.4 μM) enhanced both GPx and superoxide dismutase activities in LP in a dose-dependent manner, likely reflecting metabolic activation of the Se pathway. Comparable trends have been documented in other species; for example, Kieliszek et al. (77) and Fujs et al. (78) observed increased GPx activity in *Candida utilis* and *Candida intermedia* following exposure to SS (380 μM and 633 μM, respectively). Additional studies have shown the same dose-dependent activity at concentrations ranging from 126.6 to 780 μM in *Saccharomyces* and *Candida* (28). Furthermore, Martínez et al. (64) found that supplementation with SS (63.3 μM) elevated glutathione reductase activity in *Fructobacillus*, likely in response to increased production of glutathione disulfide generated by enhanced GPx. Our findings further support that both low and high doses of SS enhance GPx activity, while additionally demonstrating that a similar effect is achieved with an organic Se source. Contrary to our findings, previous studies have reported that GPx enzymatic upregulation can occur as a compensatory response to mild oxidative stress induced by Se exposure, serving as a signaling cue that promotes *de novo* synthesis of antioxidant proteins (28, 64, 74, 79). In our study, however, the observed increase in GPx activity is more likely attributable to efficient Se utilization, independent of the initial Se source, rather than to a stress-induced response, as intracellular ROS levels remained unchanged.

As reported by Pophaly et al. (79), LP possesses genes encoding glutathione reductase and GPx, emphasizing the importance of redox regulation in LAB, especially for protection against gastrointestinal stress, an aspect frequently applied in the industry. Accordingly, our results demonstrated that higher Se concentrations are able to increase *gpx* gene expression. Interestingly, SeMet produced a notably stronger gene expression response compared to SS. This could be related with the higher internal pool in the LP-SM250 which likely provides the necessary metabolic substrate for the significant transcriptional upregulation of antioxidant genes. Nevertheless, the gene upregulation observed in LP-SS250 highlights that Se is being converted to organic forms and accumulated, potentially acting as an endogenous reserve for selenoprotein synthesis. Although few studies have directly examined the effect of Se supplementation on *gpx* gene expression, Zhou et al. (80) demonstrated that LP exposed to H₂O₂ upregulated *gpx* and *gshr* genes, confirming that oxidative stress can drive antioxidant gene expression. Similarly, in *Rhodobacter sphaeroides,* selenite exposure led to the upregulation of superoxide dismutase probably attributed to an increase of *sodB* (74). Also, several *Lactobacillus* strains have been shown to increase *gpx* expression in response to oxidative stressors such as H₂O₂ (80–82). Notably, our findings indicate that this transcriptional response occurs even in the absence of an external oxidative stressor. Moreover, while yeast studies have shown that higher transcript levels of antioxidant enzymes do not always correspond to increased protein expression or enzymatic activity (83), our results suggest otherwise.

In the second part of our study, we investigated the effects of the Se-enriched LP (probiotic) and non-lysed CFS (postbiotic) on a Caco-2 cell model using both inorganic and organic Se sources. Regarding the probiotic approach, existing literature has primarily focused on the use of SS for Se enrichment in microorganisms [see (25, 84) for review]. However, to date, no studies have examined the effects of SeMet supplementation delivered through Se-enriched bacteria on Caco-2 cells. Moreover, regarding the postbiotic effect, no previous studies have evaluated non-lysed CFS from Se-enriched bacteria in intestinal cell cultures. Furthermore, in terms of the postbiotic approach, there is a lack of research evaluating the impact of non-lysed CFS derived from Se-enriched bacteria in intestinal cell culture models. In this context, our study addresses a relevant gap in the field by providing a comparison between Se sources and delivery formats, offering new insights into how these forms may influence cellular response in intestinal cells.

Interestingly, both Se-enriched LP and its corresponding CFS, regardless of Se source, significantly upregulated *GPX1* mRNA expression in Caco-2 cells. This finding aligns with the evidence showing that Se-enriched bacteria can modulate epithelial barrier related gene expression under oxidative injury (45, 84). Moreover, Se-enriched *L. plantarum* improved antioxidant capacity, enhanced resistance to digestive conditions, and mitigated inflammation in mice (85, 86). Furthermore, Se or Se/Zn-enriched probiotics can enhance antioxidant gene transcription and increase enzymatic biomarkers in serum of Wistar rats (87) and canine blood (88), particularly under physiological stress. Notably, our results further suggest that this regulatory effect can also occur at physiological, non-stressed conditions, potentially preparing the intestinal epithelium to better respond to subsequent injury.

It is important to highlight that, at higher concentrations, a superior response was observed in cells treated with the whole bacteria compared with CFS alone. This result likely relates to the higher Se concentration present in the whole bacterial fraction, whereas the CFS contains only excreted metabolites and does not include lysed bacteria or their intracellular content. Moreover, it should be taken into account that Se delivered through intact bacterial cells is more bioavailable, likely because the bacterial structure acts as a concentrated carrier of intracellular organic Se species or SeNPs (86). The bacterial cell wall may facilitate Se delivery and uptake (89), leading to a stronger stimulation of *GPX1* expression than that achieved by the secreted metabolites present in the CFS. Nevertheless, it is noteworthy that the CFS still induced a comparable effect despite its lower Se content, suggesting that secreted Se-containing metabolites may exert relevant biological activity even in the absence of whole bacterial cells. Such findings are particularly significant given that cell-free preparations are generally considered more stable, easier to standardize, and potentially safer than live bacteria, especially in situations where the use of viable microorganisms may be limited, such as in immunocompromised individuals or in formulations requiring long shelf life (90).

Another noteworthy aspect is that several studies have reported that high concentrations of SS are cytotoxic to intestinal epithelial cells [i.e., (19)]. Considering that bacteria use detoxification mechanisms and accumulate a portion of Se while transforming and excreting the rest (34, 64, 91), they may enable the delivery of Se at concentrations that would otherwise be toxic in its inorganic form. Moreover, in the study by Campos-Sabariz et al. (19), we observed that incubation of Caco-2 cells with SS did not induce changes in gene expression. In contrast, the present study highlights that SS-enriched bacteria, and even their corresponding CFS, can induce changes in gene expression. Additionally, the lack of significant difference between the untreated negative controls (No LP/ CFS-No LP) and the non-enriched bacterial controls (LP0/CFS-LP0) further confirms that these transcriptional changes are specifically driven by the Se enrichment rather than the presence of the probiotic or postbiotic alone.

Furthermore, an interesting result can be the difference between mRNA expression of *GPX1* and functional outcomes, as evidenced by the stable levels of GPX enzymatic activity and ROS production across all treatments. This discrepancy suggests a post-transcriptional holdup explained by the hierarchy of selenoprotein synthesis. Under non-stressed basal conditions, cells prioritize the translation of essential selenoproteins, such as thioredoxin reductase, over *GPX1*. As noted by Wang and Fu (89), while SeMet and nano-Se enhance cellular Se retention, it does not always trigger an immediate increase in GPX activity due to the highly regulated co-translational machinery required for biosynthesizing these enzymes. Since redox homeostasis was maintained in the current study, these findings suggest that LP or CFS do not negatively impact cellular function. The additional *GPX1* mRNA pool likely represents a primed state ready for future oxidative challenges. This is consistent with animal models (heat stress in canines or H_2_O_2_ exposure in mice) where functional enzymatic increases were only shown following an acute challenge (45, 85, 87).

While this study provides novel insights into the distinct bioactivity of Se-enriched *L. plantarum* and its metabolite fraction, certain limitations should be acknowledged. First, this work focused on evaluating the overall functional outcomes and antioxidant responses of the Se-enriched fractions. Therefore, the precise underlying molecular pathways and upstream signaling pathways underlying these effects remain to be fully characterized. Second, *L. plantarum* is universally recognized as a safe (GRAS) microorganism. This safety profile is supported by the ROS and GPX activity results obtained in the present study, where Se-enriched groups showed no evidence of pro-oxidant effects under basal conditions. Although both the bacterial biomass and its CFS appeared biocompatible in this model, future studies should include comprehensive cytotoxicity assessments to further support their potential therapeutic or nutraceutical applications. Finally, while the functional benefits of the non-lysed CFS were clearly demonstrated, the specific metabolites responsible for these effects remain to be identified. Future investigations incorporating detailed multiomic pathway mapping, cellular imaging approaches, and *in vivo* models will be valuable for elucidating the mechanisms involved and exploring their potential translational applications.

In summary, our results demonstrate that LP effectively utilizes both Se sources, significantly enhancing its antioxidant potential even at lower concentrations. At the transcriptional level, higher Se concentrations, mainly in the form of SeMet and consistent with the greater intracellular accumulation observed, exerted a stronger stimulatory effect on *gpx* expression. These findings suggest that increased Se availability triggers an adaptive metabolic response aimed at reinforcing antioxidant protection. Thus, our results suggest that LP actively integrates Se into a defensive metabolic framework rather than merely accumulating it passively, an adaptation that may enhance its resilience to oxidative challenges during gastrointestinal transit or industrial processing. Furthermore, by demonstrating that both live bacteria and even the non-lysed postbiotic fraction can significantly upregulate antioxidant-related genes (*GPX1*) in intestinal cells under physiological conditions, this study extends current knowledge beyond traditional biomass-focused approaches and highlights the potential of standardized Se-enriched microbial preparations for host redox modulation.

Ultimately, these findings highlight Se-enriched *L. plantarum* preparations, as both probiotics and postbiotics, as bioactive and biocompatible modulators of intestinal antioxidant gene systems capable of supporting redox homeostasis under physiological conditions. While further studies are required to understand the underlying mechanisms and validate these effects *in vivo*, our results expand current knowledge on Se-enriched LAB and support their potential application in next-generation functional foods and targeted dietary supplements aimed at mitigating gut-associated oxidative stress.

## Data Availability

The sequencing data analyzed in this study can be found in the NCBI GenBank (https://www.ncbi.nlm.nih.gov/genbank/), under accession no. NZ_CP024413.

## References

[1] Lyons M Papazyan T Surai P. Selenium in food chain and animal nutrition: lessons from nature-review. Asian Australas J Anim Sci. (2007) 20:1135–55. doi: 10.5713/ajas.2007.1135

[2] Mangiapane E Pessione A Pessione E. Selenium and selenoproteins: an overview on different biological systems. Curr Protein Pept Sci. (2014) 15:598–607. doi: 10.2174/1389203715666140608151134, 24910086

[3] Kieliszek M Błażejak S. Current knowledge on the importance of selenium in food for living organisms: a review. Molecules. (2016) 21:609. doi: 10.3390/molecules21050609, 27171069 PMC6274134

[4] Kieliszek M Serrano Sandoval SN. The importance of selenium in food enrichment processes. A comprehensive review. J Trace Elem Med Biol. (2023) 79:127260. doi: 10.1016/j.jtemb.2023.127260 37421809

[5] Avery JC Hoffmann PR. Selenium, Selenoproteins, and immunity. Nutrients. (2018) 10:1203. doi: 10.3390/nu10091203, 30200430 PMC6163284

[6] Dumont E Vanhaecke F Cornelis R. Selenium speciation from food source to metabolites: a critical review. Anal Bioanal Chem. (2006) 385:1304–23. doi: 10.1007/s00216-006-0529-8, 16830114

[7] Bai S Zhang M Tang S Li M Wu R Wan S . Effects and impact of selenium on human health, a review. Molecules. (2025) 30:50. doi: 10.3390/molecules30010050, 39795109 PMC11721941

[8] Mrvčić J Stanzer D Šolić E Stehlik-Tomas V. Interaction of lactic acid bacteria with metal ions: opportunities for improving food safety and quality. World J Microbiol Biotechnol. (2012) 28:2771–82. doi: 10.1007/s11274-012-1094-2, 22806724

[9] Johnson CC Fordyce FM Rayman MP. Symposium on ‘geographical and geological influences on nutrition’ factors controlling the distribution of selenium in the environment and their impact on health and nutrition: conference on ‘over- and undernutrition: challenges and approaches’. Proc Nutr Soc. (2010) 69:119–32. doi: 10.1017/S0029665109991807 19968907

[10] Huang Z Rose AH Hoffmann PR. The role of selenium in inflammation and immunity: from molecular mechanisms to therapeutic opportunities. Antioxid Redox Signal. (2012) 16:705–43. doi: 10.1089/ars.2011.4145, 21955027 PMC3277928

[11] Kieliszek M Błażejak S Bzducha-Wróbel A Kot AM. Effect of selenium on lipid and amino acid metabolism in yeast cells. Biol Trace Elem Res. (2018) 187:316–27. doi: 10.1007/s12011-018-1342-x, 29675568 PMC6315055

[12] Zhang Y Jin J Huang B Ying H He J Jiang L. Selenium metabolism and Selenoproteins in prokaryotes: a bioinformatics perspective. Biomolecules. (2022) 12:917. doi: 10.3390/biom12070917, 35883471 PMC9312934

[13] Lu J Holmgren A. Selenoproteins *. J Biol Chem. (2009) 284:723–7. doi: 10.1074/jbc.R800045200, 18757362

[14] Minich WB. Selenium metabolism and biosynthesis of Selenoproteins in the human body. Biochem Biokhimiia. (2022) 87:S168–77. doi: 10.1134/S0006297922140139, 35501994 PMC8802287

[15] Tangjaidee P Swedlund P Xiang J Yin H Quek SY. Selenium-enriched plant foods: selenium accumulation, speciation, and health functionality. Front Nutr. (2023) 9:962312. doi: 10.3389/fnut.2022.962312, 36815133 PMC9939470

[16] Barchielli G Capperucci A Tanini D. The role of selenium in pathologies: an updated review. Antioxidants. (2022) 11:251. doi: 10.3390/antiox11020251, 35204134 PMC8868242

[17] Rayman MP. Selenium intake, status, and health: a complex relationship. Hormones (Athens). (2020) 19:9–14. doi: 10.1007/s42000-019-00125-5, 31388899 PMC7033057

[18] Yuan S Zhang Y Dong P-Y Chen Yan Y-M Liu J Zhang B-Q . A comprehensive review on potential role of selenium, selenoproteins and selenium nanoparticles in male fertility. Heliyon. (2024) 10:e34975. doi: 10.1016/j.heliyon.2024.e34975, 39144956 PMC11320318

[19] Campo-Sabariz J Moral-Anter D Brufau MT Briens M Pinloche E Ferrer R . 2-hydroxy-(4-methylseleno)butanoic acid is used by intestinal Caco-2 cells as a source of selenium and protects against oxidative stress. J Nutr. (2019) 149:2191–8. doi: 10.1093/jn/nxz190, 31504719

[20] Campo-Sabariz J García-Vara A Moral-Anter D Briens M Hachemi MA Pinloche E . Hydroxy-Selenomethionine, an organic selenium source, increases Selenoprotein expression and positively modulates the inflammatory response of LPS-stimulated macrophages. Antioxidants. (2022) 11:1876. doi: 10.3390/antiox11101876, 36290599 PMC9598155

[21] Speckmann B Steinbrenner H. Selenium and selenoproteins in inflammatory bowel diseases and experimental colitis. Inflamm Bowel Dis. (2014) 20:1110–9. doi: 10.1097/MIB.0000000000000020, 24694793

[22] Sun Y Wang Z Gong P Yao W Ba Q Wang H. Review on the health-promoting effect of adequate selenium status. Front Nutr. (2023) 10:1136458. doi: 10.3389/fnut.2023.1136458, 37006921 PMC10060562

[23] Kasaikina MV Kravtsova MA Lee BC Seravalli J Peterson DA Walter J . Dietary selenium affects host selenoproteome expression by influencing the gut microbiota. FASEB J. (2011) 25:2492–9. doi: 10.1096/fj.11-181990, 21493887 PMC3114522

[24] Takahashi K Suzuki N Ogra Y. Effect of gut microflora on nutritional availability of selenium. Food Chem. (2020) 319:126537. doi: 10.1016/j.foodchem.2020.126537 32193059

[25] Muhammad AI Dalia AAM Hemly NIM Zainudin NN Samsudin AA. Biofortified Bacteria: the role of selenium-enriched microorganisms in enhancing animal selenium uptake—a review. J Anim Physiol Anim Nutr. (2025) 109:1298–320. doi: 10.1111/jpn.70001, 40674621

[26] Alzate A Fernández-Fernández A Pérez-Conde MC Gutiérrez AM Cámara C. Comparison of biotransformation of inorganic selenium by Lactobacillus and Saccharomyces in lactic fermentation process of yogurt and kefir. J Agric Food Chem. (2008) 56:8728–36. doi: 10.1021/jf8013519, 18729458

[27] Hachemi MA Cardoso D De Marco M Geraert P-A Briens M. Inorganic and organic selenium speciation of Seleno-yeasts used as feed additives: new insights from elemental selenium determination. Biol Trace Elem Res. (2023) 201:5839–47. doi: 10.1007/s12011-023-03633-z, 36934195 PMC10620252

[28] Kieliszek M Błażejak S Bzducha-Wróbel A Kot AM. Effect of selenium on growth and antioxidative system of yeast cells. Mol Biol Rep. (2019) 46:1797–808. doi: 10.1007/s11033-019-04630-z, 30734169

[29] Kurek E Ruszczyńska A Wojciechowski M Łuciuk A Michalska-Kacymirow M Motyl I . Bio-transformation of selenium in Se-enriched bacterial strains of *Lactobacillus casei*. Rocz Panstw Zakl Hig. (2016) 67:253–62. 27546322

[30] Liao J Wang C. Factors affecting selenium-enrichment efficiency, metabolic mechanisms and physiological functions of selenium-enriched lactic acid bacteria. J Future Foods. (2022) 2:285–93. doi: 10.1016/j.jfutfo.2022.08.001

[31] Hrdina J Banning A Kipp A Loh G Blaut M Brigelius-Flohé R. The gastrointestinal microbiota affects the selenium status and selenoprotein expression in mice. J Nutr Biochem. (2009) 20:638–48. doi: 10.1016/j.jnutbio.2008.06.009, 18829286

[32] Kieliszek M Dourou M. Effect of selenium on the growth and lipid accumulation of Yarrowia lipolytica yeast. Biol Trace Elem Res. (2021) 199:1611–22. doi: 10.1007/s12011-020-02266-w, 32632749 PMC7886723

[33] Zan L Chen Z Zhang B Zou X Lan A Zhang W . Screening, characterization and probiotic properties of selenium-enriched lactic acid bacteria. Fermentation (Basel). (2024) 10:39. doi: 10.3390/fermentation10010039

[34] Zare D Aryaee H Mirdamadi S Shirkhan F. The benefits and applications of *Lactobacillus plantarum* in food and health: a narrative review. Iran J Public Health. (2024) 53:2201–13. doi: 10.18502/ijph.v53i10.16698 39544869 PMC11557752

[35] Wei C-X Wu J-H Huang Y-H Wang X-Z Li J-Y. *Lactobacillus plantarum* improves LPS-induced Caco2 cell line intestinal barrier damage via cyclic AMP-PKA signaling. PLoS One. (2022) 17:e0267831. doi: 10.1371/journal.pone.0267831, 35639684 PMC9154120

[36] Karczewski J Troost FJ Konings I Dekker J Kleerebezem M Brummer R-JM . Regulation of human epithelial tight junction proteins by *Lactobacillus plantarum* in vivo and protective effects on the epithelial barrier. Am J Physiol Gastrointest Liver Physiol. (2010) 298:G851–9. doi: 10.1152/ajpgi.00327.2009, 20224007

[37] Liu J Shi L Ma X Jiang S Hou X Li P . Characterization and anti-inflammatory effect of selenium-enriched probiotic *Bacillus amyloliquefaciens* C-1, a potential postbiotics. Sci Rep. (2023) 13:14302. doi: 10.1038/s41598-023-40988-8, 37652982 PMC10471622

[38] Vera-Santander VE Hernández-Figueroa RH Jiménez-Munguía MT Mani-López E López-Malo A. Health benefits of consuming foods with bacterial probiotics, postbiotics, and their metabolites: a review. Molecules. (2023) 28:1230. doi: 10.3390/molecules28031230, 36770898 PMC9920731

[39] Chang HM Foo HL Loh TC Lim ETC Abdul Mutalib NE. Comparative studies of inhibitory and antioxidant activities, and organic acids compositions of postbiotics produced by probiotic *Lactiplantibacillus plantarum* strains isolated from Malaysian foods. Front Vet Sci. (2021) 7:602280. doi: 10.3389/fvets.2020.602280, 33575277 PMC7870707

[40] Adadi P Barakova NV Muravyov KY Krivoshapkina EF. Designing selenium functional foods and beverages: a review. Food Res Int. (2019) 120:708–25. doi: 10.1016/j.foodres.2018.11.029, 31000289

[41] Chen Z Lu Y Dun X Wang X Wang H. Research Progress of selenium-enriched foods. Nutrients. (2023) 15:4189. doi: 10.3390/nu15194189, 37836473 PMC10574215

[42] Zhao S Zhao X Liu Q Jiang Y Li Y Feng W . Protective effect of *Lactobacillus plantarum* ATCC8014 on acrylamide-induced oxidative damage in rats. Appl Biol Chem. (2020) 63:43. doi: 10.1186/s13765-020-00527-9

[43] Tsilingiri K Barbosa T Penna G Caprioli F Sonzogni A Viale G . Probiotic and postbiotic activity in health and disease: comparison on a novel polarised ex-vivo organ culture model. Gut. (2012) 61:1007–15. doi: 10.1136/gutjnl-2011-300971, 22301383

[44] Guan L Hu A Ma S Liu J Yao X Ye T . Lactiplantibacillus plantarum postbiotic protects against Salmonella infection in broilers via modulating NLRP3 inflammasome and gut microbiota. Poult Sci. (2024) 103:103483. doi: 10.1016/j.psj.2024.103483, 38354474 PMC10875300

[45] Izuddin WI Humam AM Loh TC Foo HL Samsudin AA. Dietary postbiotic *Lactobacillus plantarum* improves serum and ruminal antioxidant activity and upregulates hepatic antioxidant enzymes and ruminal barrier function in post-weaning lambs. Antioxidants. (2020) 9:250. doi: 10.3390/antiox9030250, 32204511 PMC7139658

[46] Zheng M Ma M Wang L Yang X Zhang Y Man C . *Lactobacillus plantarum* J26 postbiotics alleviate high-fat and high-cholesterol diet-induced hypercholesterolemia via regulating the LXRα-CYP7A1-bile acid-excretion pathway. Food Front. (2023) 4:2045–57. doi: 10.1002/fft2.310

[47] Pieniz S Okeke BC Andreazza R Brandelli A. Evaluation of selenite bioremoval from liquid culture by *Enterococcus* species. Microbiol Res. (2011) 166:176–85. doi: 10.1016/j.micres.2010.03.005, 20634050

[48] Dinu L-D Avram I Pelinescu D-R Vamanu E. Mineral-enriched postbiotics: a new perspective for microbial therapy to prevent and treat gut dysbiosis. Biomedicine. (2022) 10:2392. doi: 10.3390/biomedicines10102392, 36289654 PMC9599024

[49] Jin W Yoon C Johnston T Ku S Ji G. Production of Selenomethionine-enriched *Bifidobacterium bifidum* BGN4 via sodium selenite biocatalysis. Molecules. (2018) 23:2860. doi: 10.3390/molecules23112860, 30400218 PMC6278457

[50] Li Y Wang T Liu D Zhang C Wu W Yi H . Biotransformation of inorganic selenium into selenium nanoparticles and organic selenium by Lactiplantibacillus plantarum CXG4. Food Biosci. (2025) 65:106060. doi: 10.1016/j.fbio.2025.106060

[51] Zhang B Zhou K Zhang J Chen Q Liu G Shang N . Accumulation and species distribution of selenium in Se-enriched bacterial cells of the *Bifidobacterium animalis* 01. Food Chem. (2009) 115:727–34. doi: 10.1016/j.foodchem.2008.12.006

[52] Brufau MT Campo-Sabariz J Carné S Ferrer R Martín-Venegas R. Salmosan, a β-galactomannan-rich product, in combination with *Lactobacillus plantarum* contributes to restore intestinal epithelial barrier function by modulation of cytokine production. J Nutr Biochem. (2017) 41:20–4. doi: 10.1016/j.jnutbio.2016.11.011, 27951516

[53] Lee Y-J Choi J-H Kang K-K Sung S-E Lee S Sung M . Antioxidant and antimelanogenic activities of *Lactobacillus kunkeei* NCHBL-003 isolated from honeybees. Microorganisms. (2024) 12:188. doi: 10.3390/microorganisms12010188, 38258014 PMC10818717

[54] Brufau MT Campo-Sabariz J Bou R Carné S Brufau J Vilà B . Salmosan, a β-galactomannan-rich product, protects epithelial barrier function in Caco-2 cells infected by *Salmonella enterica* serovar enteritidis. J Nutr. (2016) 146:1492–8. doi: 10.3945/jn.116.232546, 27358412

[55] Wu R Song X Liu Q Ma D Xu F Wang Q . Gene expression of *Lactobacillus plantarum* FS5-5 in response to salt stress. Ann Microbiol. (2016) 66:1181–8. doi: 10.1007/s13213-016-1199-1

[56] Martín-Venegas R Roig-Pérez S Ferrer R Moreno JJ. Arachidonic acid cascade and epithelial barrier function during Caco-2 cell differentiation. J Lipid Res. (2006) 47:1416–23. doi: 10.1194/jlr.M500564-JLR200, 16585783

[57] Martín-Venegas R Brufau MT Mañas-Cano O Mercier Y Nonis MK Ferrer R. Monocarboxylate transporter 1 is up-regulated in Caco-2 cells by the methionine precursor DL-2-hydroxy-(4-methylthio)butanoic acid. Vet J. (2014) 202:555–60. doi: 10.1016/j.tvjl.2014.09.019, 25447800

[58] Pescuma M Gomez-Gomez B Perez-Corona T Font G Madrid Y Mozzi F. Food prospects of selenium enriched-*Lactobacillus acidophilus* CRL 636 and *Lactobacillus reuteri* CRL 1101. J Funct Foods. (2017) 35:466–73. doi: 10.1016/j.jff.2017.06.009

[59] Pophaly SD Poonam Singh P Kumar H Tomar SK Singh R. Selenium enrichment of lactic acid bacteria and bifidobacteria: a functional food perspective. Trends Food Sci Technol. (2014) 39:135–45. doi: 10.1016/j.tifs.2014.07.006

[60] Rother M. "Selenium metabolism in prokaryotes". In: Hatfield DL Berry MJ Gladyshev VN, editors. Selenium: Its Molecular Biology and Role in Human Health. New York: Springer (2012). p. 457–70.

[61] Luo L Hou X Yi D Deng G Wang Z Peng M. Selenium-enriched microorganisms: metabolism, production, and applications. Microorganisms. (2025) 13:1849. doi: 10.3390/microorganisms13081849, 40871353 PMC12388498

[62] Nie X Yang X He J Liu P Shi H Wang T . Bioconversion of inorganic selenium to less toxic selenium forms by microbes: a review. Front Bioeng Biotechnol. (2023) 11:1167123. doi: 10.3389/fbioe.2023.1167123, 36994362 PMC10042385

[63] Adnan F Jalil A Ahmed T Rahman A Dawood N Haider G . TRAP transporter TakP: a key player in the resistance against selenite-induced oxidative stress in *Rhodobacter sphaeroides*. Microbiol Res. (2021) 252:126828. doi: 10.1016/j.micres.2021.126828, 34543948

[64] Martínez FG Moreno-Martin G Pescuma M Madrid-Albarrán Y Mozzi F. Biotransformation of selenium by lactic acid Bacteria: formation of Seleno-nanoparticles and Seleno-amino acids. Front Bioeng Biotechnol. (2020) 8:506. doi: 10.3389/fbioe.2020.00506, 32596220 PMC7303280

[65] Nasim MJ Zuraik MM Abdin AY Ney Y Jacob C. Selenomethionine: a pink trojan redox horse with implications in aging and various age-related diseases. Antioxidants. (2021) 10:882. doi: 10.3390/antiox10060882, 34072794 PMC8229699

[66] Schrauzer GN. The nutritional significance, metabolism and toxicology of selenomethionine. Adv Food Nutr Res. (2003) 47:73–112. doi: 10.1016/s1043-4526(03)47002-2 14639782

[67] Andreoni V Luischi MM Cavalca L Erba D Ciappellano S. Selenite tolerance and accumulation in the Lactobacillus species. Ann Microbiol. (2000) 50:77–88.

[68] Calomme M Hu J Van den Branden K Vanden Berghe DA. Seleno-lactobacillus. An organic selenium source. Biol Trace Elem Res. (1995) 47:379–83. doi: 10.1007/BF02790140, 7779573

[69] Heider J Böck A. Selenium metabolism in micro-organisms. Adv Microb Physiol. (1993) 35:71–109. doi: 10.1016/s0065-2911(08)60097-1, 8310883

[70] Kessi J Ramuz M Wehrli E Spycher M Bachofen R. Reduction of selenite and detoxification of elemental selenium by the phototrophic bacterium *Rhodospirillum rubrum*. Appl Environ Microbiol. (1999) 65:4734–40. doi: 10.1128/AEM.65.11.4734-4740.1999, 10543779 PMC91637

[71] Plateau P Saveanu C Lestini R Dauplais M Decourty L Jacquier A . Exposure to selenomethionine causes selenocysteine misincorporation and protein aggregation in *Saccharomyces cerevisiae*. Sci Rep. (2017) 7:44761. doi: 10.1038/srep44761, 28303947 PMC5355996

[72] Zhu H Zhou Y Qi Y Ji R Zhang J Qian Z . Preparation and characterization of selenium enriched-*Bifidobacterium longum* DD98, and its repairing effects on antibiotic-induced intestinal dysbacteriosis in mice. Food Funct. (2019) 10:4975–84. doi: 10.1039/c9fo00960d, 31343650

[73] Chen Y Li Q Xia C Yang F Xu N Wu Q . Effect of selenium supplements on the antioxidant activity and nitrite degradation of lactic acid bacteria. World J Microbiol Biotechnol. (2019) 35:61. doi: 10.1007/s11274-019-2609-x, 30919142

[74] Bebien M Chauvin J-P Adriano J-M Grosse S Verméglio A. Effect of selenite on growth and protein synthesis in the phototrophic bacterium *Rhodobacter sphaeroides*. Appl Environ Microbiol. (2001) 67:4440–7. doi: 10.1128/AEM.67.10.4440-4447.2001, 11571140 PMC93187

[75] Chen C Tian J Zhou J Ni X Lei J Wang X. Bacterial growth, morphology, and cell component changes in Herbaspirillum sp. WT00C exposed to high concentration of selenate. J Basic Microbiol. (2020) 60:304–21. doi: 10.1002/jobm.201900586, 31898337

[76] Labunskyy VM Hatfield DL Gladyshev VN. Selenoproteins: molecular pathways and physiological roles. Physiol Rev. (2014) 94:739–77. doi: 10.1152/physrev.00039.2013, 24987004 PMC4101630

[77] Kieliszek M Bierla K Jiménez-Lamana J Kot AM Alcántara-Durán J Piwowarek K . Metabolic response of the yeast *Candida utilis* during enrichment in selenium. Int J Mol Sci. (2020) 21:5287. doi: 10.3390/ijms21155287, 32722488 PMC7432028

[78] Fujs S Gazdag Z Poljsak B Stibilj V Milacic R Pesti M . The oxidative stress response of the yeast *Candida intermedia* to copper, zinc, and selenium exposure. J Basic Microbiol. (2005) 45:125–35. doi: 10.1002/jobm.200410480, 15812857

[79] Pophaly SD Poonam S Pophaly SD Kapila S Nanda DK Tomar SK . Glutathione biosynthesis and activity of dependent enzymes in food-grade lactic acid bacteria harbouring multidomain bifunctional fusion gene (gshF). J Appl Microbiol. (2017) 123:194–203. doi: 10.1111/jam.13471, 28403558

[80] Zhou Y Gong W Xu C Zhu Z Peng Y Xie C. Probiotic assessment and antioxidant characterization of *Lactobacillus plantarum* GXL94 isolated from fermented chili. Front Microbiol. (2022) 13:997940. doi: 10.3389/fmicb.2022.997940, 36466645 PMC9712218

[81] Gao Y Liu Y Ma F Sun M Mu G Tuo Y. Global transcriptomic and proteomics analysis of *Lactobacillus plantarum* Y44 response to 2,2-azobis(2-methylpropionamidine) dihydrochloride (AAPH) stress. J Proteome. (2020) 226:103903. doi: 10.1016/j.jprot.2020.103903, 32682107

[82] Zhai Z Yang Y Wang H Wang G Ren F Li Z . Global transcriptomic analysis of *Lactobacillus plantarum* CAUH2 in response to hydrogen peroxide stress. Food Microbiol. (2020) 87:103389. doi: 10.1016/j.fm.2019.103389, 31948630

[83] Cyrne L Martins L u Fernandes L Marinho HS. Regulation of antioxidant enzymes gene expression in the yeast *Saccharomyces cerevisiae* during stationary phase. Free Radic Biol Med. (2003) 34:385–93. doi: 10.1016/S0891-5849(02)01300-X, 12543254

[84] Yang J Yang H. Recent development in se-enriched yeast, lactic acid bacteria and bifidobacteria. Crit Rev Food Sci Nutr. (2023) 63:411–25. doi: 10.1080/10408398.2021.1948818, 34278845

[85] Kang S Li R Jin H You HJ Ji GE. Effects of selenium- and zinc-enriched *Lactobacillus plantarum* SeZi on antioxidant capacities and gut microbiome in an ICR mouse model. Antioxidants. (2020) 9:1028. doi: 10.3390/antiox9101028, 33096847 PMC7589369

[86] Wang Y Ma H Li H Huang Y Tang Y Tang X . Selenium-enriched Lactiplantibacillus plantarum ZZU 8–12 regulates intestinal microbiota and inhibits acute liver injury. Probiotics Antimicrob Proteins. (2025) 17:5095–107. doi: 10.1007/s12602-025-10459-9, 39875778

[87] Malyar RM Li H Liu D Abdulrahim Y Farid RA Gan F . Selenium/zinc-enriched probiotics improve serum enzyme activity, antioxidant ability, inflammatory factors and related gene expression of Wistar rats inflated under heat stress. Life Sci. (2020) 248:117464. doi: 10.1016/j.lfs.2020.117464, 32097667

[88] Ren Z Zhao Z Wang Y Huang K. Preparation of selenium/zinc-enriched probiotics and their effect on blood selenium and zinc concentrations, antioxidant capacities, and intestinal microflora in canine. Biol Trace Elem Res. (2011) 141:170–83. doi: 10.1007/s12011-010-8734-x, 20563665

[89] Wang Y Fu L. Forms of selenium affect its transport, uptake and glutathione peroxidase activity in the Caco-2 cell model. Biol Trace Elem Res. (2012) 149:110–6. doi: 10.1007/s12011-012-9395-8, 22451375

[90] Ma L Tu H Chen T. Postbiotics in human health: a narrative review. Nutrients. (2023) 15:291. doi: 10.3390/nu15020291, 36678162 PMC9863882

[91] Ataollahi F Amirheidari B Amirheidari Z Ataollahi M. Clinical and mechanistic insights into biomedical application of se-enriched probiotics and biogenic selenium nanoparticles. Biotechnol Lett. (2025) 47:18. doi: 10.1007/s10529-024-03559-z, 39826010

